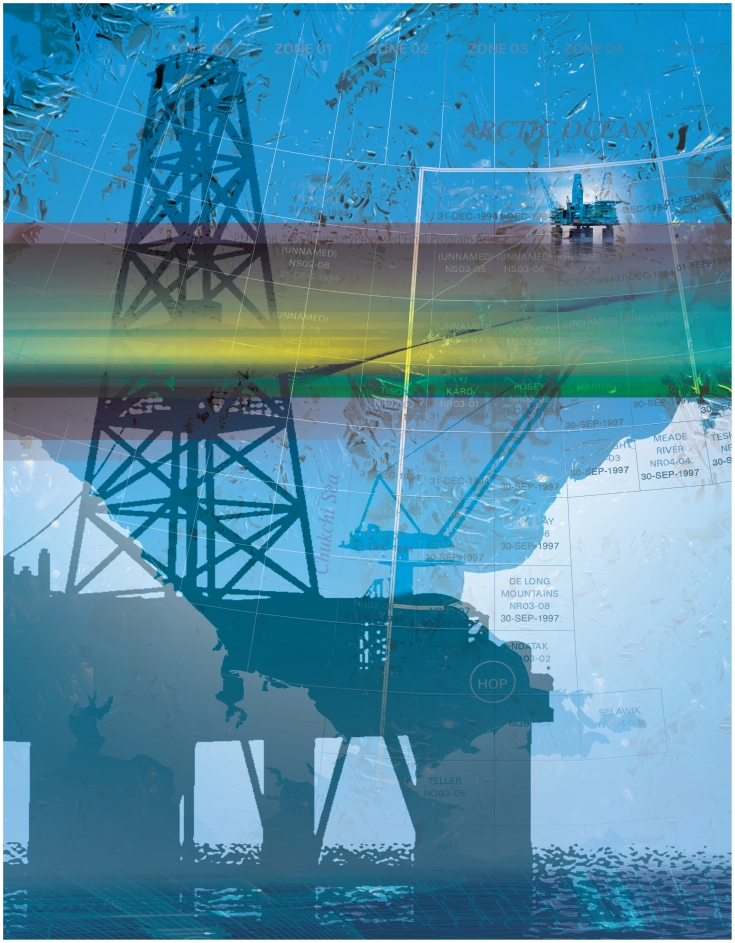# Cold Hard Cache: The Arctic Drilling Controversy

**DOI:** 10.1289/ehp.118-a394

**Published:** 2010-09

**Authors:** Charles W. Schmidt

**Affiliations:** **Charles W. Schmidt**, MS, an award-winning science writer from Portland, ME, has written for *Discover Magazine*, *Science*, and *Nature Medicine*

On 27 May 2010, with crude oil gushing into the Gulf of Mexico after the explosion of BP’s *Deepwater Horizon* oil rig, the Obama administration announced it would pause offshore drilling plans in the Arctic Ocean, one of the planet’s most pristine ecosystems.^1^ Hailed by environmental groups, the decision was a major setback to the oil industry, which was gearing up to tap what’s expected to be vast amounts of oil and gas lying under the Arctic’s treacherous waters, wher e sustained winds blow at 30 to 50 miles per hour, and menacing chunks of floating “pack ice,” some hundreds of feet wide and dozens of feet thick, threaten marine traffic.

With shallow-water, near-shore reserves increasingly tapped out in the Gulf of Mexico, oil companies are being forced into more challenging terrain to sustain domestic energy production. That means pushing into much deeper geology in the Gulf of Mexico—much of it more than a mile underwater—and also into ecologically fragile locations off the coast of Alaska.

In decades past, oil companies didn’t prioritize the offshore Arctic for development because drilling there was too expensive. Oil prices simply weren’t high enough to sustain production windows limited to just a few months in summer, when thawing seas make drilling possible, explains Layla Hughes, an attorney and senior program officer for Arctic oil, gas, and shipping policy at the WWF in Juneau. But as economies in China, India, and Brazil have grown, global fuel demands have risen accordingly,^2^ driving prices higher and making offshore Arctic development economically viable. “Prices started going up in the late nineties, and they’re projected to stay high,” Hughes says.

Oil companies now have to prove they can extract those resources safely, without compromising an ecosystem that might never recover from the effects of a large blowout. Dana Wetzel, an ecotoxicologist at Mote Marine Laboratory with 10 years’ research experience in the Arctic, says oil degradation depends largely on temperature. When exposed to frigid water, oil turns quickly into a thick, tarry substance that microbes can’t easily degrade.

The 1989 *Exxon Valdez* spill, which occurred roughly 400 miles south of the Arctic Circle, likely underestimates the full environmental impact of a blowout even farther to the north. “The *Valdez* was a horrible spill in a rich marine ecosystem, but you didn’t have the oiling of the sea ice,” Wetzel says. “If you taint sea ice with oil, you’re never going to get rid of it. And those ice floes are home to walruses, seals, and polar bears. Many people survive up there by subsistence hunting, and to contaminate their food is one of the worst moral blows you can deliver to those communities.”^3^

## Exploration Interrupted

The Alaska Outer Continental Shelf (OCS), a swath of submerged territory under U.S. federal control—contains an estimated 23% of the nation’s technically recoverable^4^ oil (26.65 billion barrels) and 21% of total technically recoverable natural gas (132.06 trillion ft^3^), according to the most recent comprehensive assessment by the U.S. Minerals Management Service (MMS),^5^ which in June 2010 was reorganized and given a new name: the Bureau of Ocean Energy Management, Regulation, and Enforcement, or BOEMRE.^6^ These projected resources were calculated based on assumptions about the nature and extent of geological formations in the area, according to Richard Ranger, senior policy advisor at the American Petroleum Institute in Washington, DC. If borne out by exploratory drilling—which would confirm estimated oil volumes and their projected financial value—these estimates put the Alaska OCS second only to the Gulf of Mexico in terms of future domestic yield.

The federal government manages OCS oil and gas resources off Alaska and also off the Gulf of Mexico and along the Pacific and Atlantic coasts using a multistage process. The Department of the Interior first decides which areas are available for drilling, and then it sells leasing rights to those areas to the highest bidder. With Lease Sale 193, Royal Dutch Shell, PLC, which is the only company that was prepared to drill this year in the Alaska OCS, paid $2.2 billion for the rights to explore in the Chukchi Sea, which lies west of Barrow, the state’s northernmost city.^7^

That 2008 purchase allowed Shell to use seismic technology to conduct geophysical searches for oil and gas in the Chukchi and also to submit an exploration plan detailing how the company would drill wells to confirm if petroleum was present in suspected locations. What was then the MMS reviewed Shell’s exploration plan and in December 2009 gave the company the green light for three exploratory wells in the Chukchi and two more in the adjoining Beaufort Sea to the east of Barrow, where Shell had purchased exploration rights in a prior lease sale, according to Shell spokesman Curtis Smith.

But after the *Deepwater Horizon* oil rig exploded on April 20, offshore operations throughout U.S. waters came under intense scrutiny. Prior to that disaster, both oil companies and the MMS could promote Arctic development on the basis of what they each claimed was a history of safe drilling in the Gulf of Mexico. The MMS deemed the likelihood of an Arctic blowout remote on the basis of its own research showing that “four wells out of fourteen thousand studied [in the Gulf] had undergone a blowout scenario, with the largest incident releasing some two hundred barrels of oil,” says BOEMRE spokesman John Callahan.

Whether the estimated 4.9 million barrels (205.8 million gallons) of oil released during the recent Gulf spill^8^ supersedes that safety record is debatable. What is clear, however, is that the spill created new anxieties about drilling in the offshore Arctic, where pack ice, storms, and intense winter cold could severely hamper efforts to contain a potential blowout.

On May 27, just as a month-long moratorium on drilling was set to expire, the administration extended it for deepwater operations in the Gulf of Mexico for an additional six months, claiming the pause was necessary to ensure oil companies working at extreme depths had adequate spill prevention and response capabilities.^1^ The administration also announced that applications for permits to drill in the Alaska OCS wouldn’t be considered until 2011 at the earliest. “[Applications for permits to drill] are the final approval necessary before drilling can occur under a previously approved exploration plan,” explains Peter Van Tuyn, an environmental lawyer based in Anchorage, who represents conservation groups and local indigenous populations.

After the federal suspension, Shell’s exploration plans took another hit on July 21, when the Alaska Federal District Court ruled the MMS hadn’t sufficiently considered environmental threats posed by drilling activities associated with Lease Sale 193.^9^ Under the National Environmental Policy Act, the MMS is required to conduct an environmental impact statement for each of its OCS lease sales. But siding with the Native Village of Point Hope, a tribal government residing on the Chukchi coast, in its litigation against Interior secretary Kenneth Salazar, the court held that in the case of Lease Sale 193 the agency’s environmental impact statement had failed to analyze the potential impacts of natural gas development (it had addressed only oil development. It also had failed to determine if “missing scientific information” (relating mainly to polar bear and walrus ecology, Van Tuyn says) was “relevant or essential” to protecting natural resources from drilling operations. The court therefore issued a cease-and-desist order against all pending activities under the lease sale, effectively blocking Shell from drilling in the Chukchi until BOEMRE revises its environmental assessment.

Smith admits these setbacks create uncertainty for the company, which he says has $3.5 billion invested toward its OCS operations in Alaska, including not just the value of its lease purchase but also oil spill response infrastructure, operational readiness, seismic investigations, and research. “We’re unsure how [the July 21 decision] might impact our aspirations to drill in 2011,” Smith says. “While the ruling is disappointing, the judge did not vacate the lease sale, and his specific concerns can be remedied expeditiously provided the BOEMRE completes the work in question.” In response, Callahan would say only that the agency is reviewing the Alaska court’s decision carefully and deciding how best to comply with it.

## The Argument for Drilling

Chuck Clusen, director of the Natural Resources Defense Council’s Alaska project, describes Shell as the biggest offshore operator in the area by far. “Shell’s at the leading edge, and the other companies are hanging back waiting for it to start so they can see what happens and then act on their own,” he says. Other companies with preliminary operations in the Alaska OCS include Chevron, Conoco Philips, Statoil (a Norwegian company), ExxonMobil, and BP, according to Ranger.

BP has spent over a decade developing its controversial Liberty Project, which aims to extract 100 million barrels of oil from under the Beaufort Sea. The company was planning to access the oil with an “extended reach” horizontal well, drilled from a gravel island built three miles offshore in Prudhoe Bay. Digging first two miles down, and then six to eight miles out horizontally to the oil reservoir, this would be the longest extended-reach well ever created, Ranger says.

Yet in the wake of the *Deepwater Horizon* disaster, BP’s offshore plans in Alaska appear to be on hold. The company has yet to submit an application for a permit to drill in the Beaufort Sea, Callahan says, and according to Ranger, BP has indicated it is likely to delay the project.

As offshore Arctic drilling waits to go forward, the essential question is whether it can be done safely. The petroleum industry insists the answer is yes. Ranger claims drilling depths currently being considered in the U.S. Arctic don’t exceed 500 feet, unlike the 5,000 depths or more in the Gulf of Mexico. Moreover, oil flows are much less pressurized in the region, he says. While the Macondo Prospect (the geological site of the *Deepwater Horizon* rig) gushes oil at a flow rate of 15,000 psi, Smith says historical data put expected flow rates in the Alaska OCS at a much lower 6,000 psi. That figure—produced by Shell during prior exploratory drilling in the Chukchi and Beaufort seas, according to Smith—has not been confirmed by external experts, and Callahan did not respond to inquiries about external review of Shell’s wellhead pressure estimate.

Nevertheless, lower pressure is held up as a key argument for the safety of Arctic drilling. According to Ranger, companies studying potential projects in the U.S. Arctic believe shallow waters will make it easier for divers and submersibles to respond to a potential blowout, while lower pressures will make a blowout less likely overall. In a May 14 letter to former MMS director S. Elizabeth Birnbaum, Shell’s president, Marvin Odum, took a similar stand. “Due to the difference in expected down-hole pressure of the Macondo well versus our planned 2010 wells, our margin to safely operate in Alaska is much greater than that experienced by *Deepwater Horizon*,” he wrote.^10^

In the letter, Odum argued that drilling muds—slurries of clay and other minerals mixed with water or oil that modulate oil flows in the pipe connecting the wellhead at the seafloor to the drill rig at the surface—would be heavy enough to contain a low-pressure blowout. He also referenced other safety features such as cement liners that strengthen the casing pipe, which extends downward from the wellhead to the oil reservoir deep underground. A series of interconnected pipes, the casing is analogous to a telescope, with a wide end at the surface that narrows as each additional pipe goes through the bore, penetrating into the geology below. Each pipe is secured with cement, Ranger explains, and that’s what ensures the integrity of the well (indeed, an inadequate cement job compromised by skimping on costs was one of the fatal flaws of the destroyed BP well^11^).

Still, Ranger admits responders would have a limited ability to contain a blowout should it occur. “The laws of physics and chemistry make it very difficult to remove one liquid, in this case oil, from another, meaning water,” he says. “All on-water oil-spill response is imperfect, and our ability to recover oil mechanically ranges from ten percent to thirty percent. Those are the realities.”

## Breaking Down the Response Plan

Arctic drilling is only viable, Hughes says, from July through late September or October. Each year the sea ice first starts separating from shore in April, creating a steadily widening stretch of open water called a lead. By July, open water dominates in the region, although portions still freeze over occasionally, and pack ice can still threaten drilling operations. Come November, the region has completely refrozen.

In a worst-case scenario, a blowout would occur in late fall, producing an oil gusher blocked at the surface by newly formed ice, says Rick Steiner, a professor of marine conservation at the University of Alaska in Anchorage. Inaccessible to skimmers, booms, and other mechanical recovery tools, oil would flow under the frozen surface and spread throughout the region. “There would be nothing you could do about it until the ice goes out again in June,” Steiner emphasizes. “The oil would be in evidence for decades. It could fundamentally restructure the Arctic Ocean ecosystem, which is already threatened by damage from climate change.”

Smith says that in the event of a major spill, the company would deploy its “world-class, on-site oil-response kit,” which includes booms, skimmers, helicopters, support vessels, and other infrastructure, all floating next to the drill site and ready for mobilization within an hour. He also claims Shell could drill a relief well within 30 days using an “ice-ready” drill rig that can work late into the winter season. The spill response plan has been reviewed and approved by the U.S. Coast Guard, Smith says. “We’re also at pre-engineering stages on a containment dome developed specifically for Arctic conditions,” he adds.

Yet in a blog posting dated May 26, Clusen wrote that Shell offers no explanation for why such a dome would be more successful than the one that failed in the Gulf. “Although much shallower, the [proposed] wells in the Arctic are sufficiently cold to produce the methane hydrate crystals that formed in the Gulf and clogged the hole at the top through which the oil was supposed to be removed by a pipe to the sea surface,” he wrote.^12^

Moreover, Hughes disputes the notion that a relief well could be drilled in less than 30 days, and claims current efforts to drill such wells at the Macondo Prospect—in their 105th day at the time of this writing—bolster her case. Patrick Lewis, officer for responsible industry at the WWF’s International Arctic Programme in Oslo, Norway, adds there’s no way the Alaskan infrastructure could handle a large offshore spill response. Where the *Deepwater Horizon* response at one point involved more than 5,300 vessels, 120 aircraft, 4.27 million feet of boom, 1.8 million gallons of dispersant, and 42,000 people,^13^ all deployed from a highly developed coastline, the Chukchi and Beaufort coastlines contain no road system, essentially no port facilities, and few airports. In his May 26 blog entry, Clusen wrote that the nearest airports are in Barrow and Point Hope (100 and 150 miles, respectively, from Shell’s proposed drill sites); the nearest U.S. Coast Guard station is in Kodiak, 1,000 miles away.^12^

Moreover, it’s unlikely that chemical dispersants used to break oil into minute particles that undergo more rapid microbial degradation underwater would be as effective in the Alaska OCS as they were in the Gulf of Mexico, according to Carys Mitchelmore, an associate professor at the University of Maryland’s Chesapeake Biological Laboratory. How well dispersants work depends in part on temperature, she says, such that applications would be more effective at the wellhead, where spewing oil is hot. But after bubbling just a few meters toward the surface in frigid water, oil thickens into a more viscous form that doesn’t respond to dispersants as readily as more fluid oil.

In Steiner’s view, Shell and the other companies proposing to work in the offshore Arctic need to develop better blowout risk assessments, prevention plans, and response measures, reviewed not just by BOEMRE but also by other independent bodies such as the National Academy of Sciences or the National Academy of Engineering. “The companies just haven’t gone through the hundred-and-one ways a blowout could occur, and in my opinion, they haven’t sufficiently articulated their plans for drilling relief wells,” he says.

Callahan responds, “The Alaska Region [of BOEMRE] can confirm that it reviewed Shell’s contingency plan and found it adequate for the time it was issued. However, in light of the BP oil spill in the Gulf and new requirements for the plans, we will be reviewing the adequacy of the current version of the project’s spill plan.”

Asked about the impact of the Gulf spill on BOEMRE policies with respect to Arctic drilling, Callahan says, “We must raise the bar for offshore oil and gas operations . . . holding them to the highest safety standards to ensure they follow the law rather than cut corners. The stronger regulatory structure, tougher safety requirements, and new leadership we are putting in place will bring about fundamental changes to how our nation oversees oil and gas operations.”

## Choosing a Future

Environmentalists interviewed for this story seem resigned to the notion that drilling on the Alaska OCS is likely at some point. “There’s a lot of oil up there, and the state of Alaska desperately wants to go forward on this,” Clusen says. Opinions among native groups are divided, adds Jonny Jemming, a Barrow lawyer who represents the North Slope Borough, an Inupiat-led municipal government in northern Alaska. Although many Inupiat, or native people of Alaska, rely in large part on marine subsistence for their dietary needs, their native corporations are also heavily contracted with oil industries in the Alaska OCS, he says.

Jemming says it’s not fair to say that all Inupiat are against offshore drilling. “There’s a realization that these decisions are ultimately made far outside our control,” he says. “Oil operations are part of the socioeconomic health of the region. But from what we have seen, the technologies for oil spill mitigation aren’t adequate to minimize the risk from these operations. What we have been asking for are the highest safety standards possible and that they be implemented before development takes place. We see this as reasonable ocean management.”

Still, that raises a difficult question: What would an offshore drilling plan in the Arctic need to demonstrate before it’s seen as reasonable? The only logical conclusion is that it would show either that a blowout would never occur or that it wouldn’t pose an unacceptable risk to the region’s fragile ecology. That sets a high bar, and the oil industry will have to show it can meet it.

Steiner adds that although offshore drilling has broad support in Alaska, the decision to go forward with it involves fundamental choices for society. “Do we continue our devastating industrial expansion into one of the last, pristine wild areas in the world, extract and use the billions of tons of fossil carbon energy there, and further degrade the environment of the region and the world?” he asks. “Or do we choose another, sustainable future?”

**The International Component**

Offshore Arctic development also has an international component, given that seven circumpolar nations—the United States, Russia, Canada, Norway, Greenland, Iceland, and Sweden—have claims to submerged territory in the region that could hold as much as of 25% of the world’s remaining petroleum reserves. In one closely watched operation, the Scottish company Cairn Energy is drilling exploration wells in the Davis Straits, an icy stretch between Greenland and Baffin Island. Statoil already drills for natural gas now in the Barents Sea at a point roughly 90 miles northwest of Hammerfest, Norway.

Patrick Lewis of the WWF International Arctic Programme says very little of the accessible Arctic sea floor is unclaimed by one country or another. “And there’s a complete absence of international regulation over the oil and gas industries,” he says. “While other global sectors, such as shipping, are regulated at the international level, the petroleum sector still depends on [national-level] regulation, which is frequently inconsistent across regions and poorly administered.”

Therefore, oil companies may choose to operate in areas where rules governing environmental safety—or enforcement of those rules—is more lax. “Unfortunately,” Lewis says, “the consequences of a major spill may well affect more than just one jurisdiction.”

## Figures and Tables

**Figure:**